# Induced Bias Due to Crossover Within Randomized Controlled Trials in Surgical Oncology: A Meta-regression Analysis of Minimally Invasive versus Open Surgery for the Treatment of Gastrointestinal Cancer

**DOI:** 10.1245/s10434-017-6210-y

**Published:** 2017-11-06

**Authors:** George Garas, Sheraz R. Markar, George Malietzis, Hutan Ashrafian, George B. Hanna, Emmanouil Zacharakis, Long R. Jiao, Athanassios Argiris, Ara Darzi, Thanos Athanasiou

**Affiliations:** 1 Surgical Epidemiology Unit, Department of Surgery and Cancer, Imperial College London, St. Mary’s Hospital, London, UK; 2 Department of Surgical Research and Innovation, The Royal College of Surgeons of England, London, UK; 3 Department of Surgery and Cancer, Imperial College London, Hammersmith Hospital, London, UK; 4 Department of Medical Oncology, Thomas Jefferson University, Philadelphia, PA USA; 5 Health Technology Assessment Committee, National Institute of Health and Care Excellence, London, UK

## Abstract

**Background:**

Randomized controlled trials (RCTs) inform clinical practice and have provided the evidence base for introducing minimally invasive surgery (MIS) in surgical oncology. Crossover (unplanned intraoperative conversion of MIS to open surgery) may affect clinical outcomes and the effect size generated from RCTs with homogenization of randomized groups.

**Objectives:**

Our aims were to identify modifiable factors associated with crossover and assess the impact of crossover on clinical endpoints.

**Methods:**

A systematic review was performed to identify all RCTs comparing MIS with open surgery for gastrointestinal cancer (1990–2017). Meta-regression analysis was performed to analyze factors associated with crossover and the influence of crossover on endpoints, including 30-day mortality, anastomotic leak rate, and early complications.

**Results:**

Forty RCTs were included, reporting on 11,625 patients from 320 centers. Crossover was shown to affect one in eight patients (mean 12.6%, range 0–45%) and increased with American Society of Anesthesiologists score (*β* = + 0.895; *p* *=* 0.050). Pretrial surgeon volume (β = − 2.344; *p* *=* 0.037), composite RCT quality score (*β* = − 7.594; *p* *=* 0.014), and site of tumor (*β* = − 12.031; *p* *=* 0.021, favoring lower over upper gastrointestinal tumors) showed an inverse relationship with crossover. Importantly, multivariate weighted linear regression revealed a statistically significant positive correlation between crossover and 30-day mortality (*β* = + 0.125; *p* *=* 0.033), anastomotic leak rate (*β* = + 0.550; *p* *=* 0.004), and early complications (*β* = + 1.255; *p* *=* 0.001), based on intention-to-treat analysis.

**Conclusions:**

Crossover in trials was associated with an increase in 30-day mortality, anastomotic leak rate, and early complications within the MIS group based on intention-to-treat analysis, although our analysis did not assess causation. Credentialing surgeons by procedural volume and excluding high comorbidity patients from initial trials are important in minimizing crossover and optimizing RCT validity.

**Electronic supplementary material:**

The online version of this article (10.1245/s10434-017-6210-y) contains supplementary material, which is available to authorized users.

Randomized controlled trials (RCTs) provide the highest level of evidence informing clinical practice, and provide the basis for national and international guidance of disease-treatment algorithms and protocols.^1^ Several RCTs have suggested benefits in short-term endpoints from minimally invasive surgery (MIS) for gastrointestinal cancer,^2^–^5^ which has led to substantial growth in the adoption of these techniques and incorporation into national and international guidelines.^6^

Adherence to the tested intervention is a fundamental principle of scientific investigation. Crossover (unplanned intraoperative conversion of MIS to open surgery) can have important consequences on trial validity, with large-scale, uncontrolled crossover potentially invalidating trial results.^7^ This is frequently the result of a combination of mechanisms, which include disruption of randomization, bias, systematic error, and loss of statistical power. Crossover can thus convert the trial from randomized to a hybrid of randomized and observational study with survival, the primary endpoint in surgical oncology trials, being compromized.^8^

The primary objective of this systematic review was to evaluate the influence of crossover within RCTs regarding MIS for gastrointestinal cancer on short-term endpoints and overall survival. The secondary objective was to identify potentially modifiable factors that significantly affect the incidence of crossover within these RCTs.

## Methods

### Search Strategy and Selection Criteria

The study was executed and reported in accordance with the Preferred Reporting Items for Systematic Reviews and Meta-Analyses (PRISMA) statement.^9^ All studies published between 1990 and 2017 without language restriction and reporting on MIS versus open treatment modality in patients undergoing surgical treatment for gastrointestinal cancer were identified. The MEDLINE, EMBASE, Google Scholar, Cochrane, PsycINFO, and ERIC electronic databases were searched (latest search 3 September 2017) using the following medical subject heading (MeSH) terms: ((“randomized controlled trial”[Publication Type] OR “randomized controlled trials as topic”[MeSH Terms] OR “randomised controlled trial”[All Fields] OR “randomized controlled trial”[All Fields]) AND (“Surg Oncol”[Journal] OR (“surgical”[All Fields] AND “oncology”[All Fields]) OR “surgical oncology”[All Fields] OR “J Surg Oncol”[Journal] OR (“surgical”[All Fields] AND “oncology”[All Fields]) OR “surgical oncology”[All Fields])) AND Clinical Trial[All Fields]). The ‘related articles’ function and reference list of each of the identified publications was used to widen the literature search. Any relevant review articles were also screened.

For inclusion in the analysis, studies had to be RCTs that compared MIS versus open surgery for the treatment of gastrointestinal cancer. Furthermore, studies had to report the endpoints of interest (described below) and comprise an adult patient group (> 18 years). All trials had to be registered. Studies were excluded if they reported a previously published dataset (in which case the most recent publication was included) and/or if they primarily investigated adjuvant (or neoadjuvant) treatments (e.g. chemotherapy and/or radiotherapy) as opposed to the type of surgical approach (i.e. MIS vs. the open approach).

### Data Extraction

Two reviewers (GG and SRM) independently assessed the articles and relevant data were extracted without cross-referencing. Any conflicts in data extraction or quality assessment were resolved by the senior authors (AD and TA) prior to analysis. The parameters and endpoints captured are described below. The quality of the included studies was assessed using a recently introduced tool for evaluating the rigor of surgical RCTs, and a composite quality score was computed for each study, taking into account the Jadad and risk-of-bias scores.^10^ A composite quality score > 3 was defined as a threshold of rigor (electronic supplementary Table S1).^11^

### Variables

The following demographic data were extracted: study type, patient numbers, year and country of publication, tumor site, disease stage, American Society of Anesthesiologists (ASA) score, treatments compared, number of centers participating in the trial, pretrial surgeon volume/experience to be allowed to enter each trial, Jadad score, risk-of-bias score, composite quality score, and presence of crossover (including percentage, time it occurred [i.e. pre- vs. intraoperative], and reasons given for it).

### Endpoints

Endpoints studied were 30-day mortality, 30-day (early) complications (specifically pulmonary complications and anastomotic leak rate), length of stay, and overall survival.

### Factors Affecting the Incidence of Crossover

The measure of crossover for each study was defined as the percentage of patients who crossed over from one study arm to the other. The effect of number of centers, pretrial surgeon volume, sample size, site of cancer [upper (esophageal and gastric) versus lower (colonic and rectal) gastrointestinal tumors, and further subdivision of lower gastrointestinal tumors into colonic and rectal], ASA score (3 + 4 vs. 1 + 2), disease stage (III + IV vs. I + II), and composite quality score of the RCT were studied. Subsequently, univariate and multivariate linear regression models were utilized to evaluate the association between crossover and the above parameters. The level of significance permitting multivariate analysis inclusion was set at *p* < 0.05.

### Crossover and Study Endpoints

Meta-regression analysis was performed to quantitatively assess the impact of (1) composite quality score, (2) number of participating centers, (3) pretrial surgeon volume, (4) sample size, (5) ASA score, and (6) crossover on the overall effect for each endpoint (30-day mortality, anastomotic leak rate, pulmonary complications, length of stay, and early complications). All variables were checked for interaction and multicollinearity. The significance level was set at *p* < 0.05.

The study was performed in line with Cochrane recommendations, following the Meta-analysis Of Observational Studies in Epidemiology (MOOSE) group guidelines (electronic supplementary Table S2)^12^ using the statistical software STATA 14 (StataCorp LP, College Station, TX, USA).

## Results

### Selected Studies

**Fig. 1:**
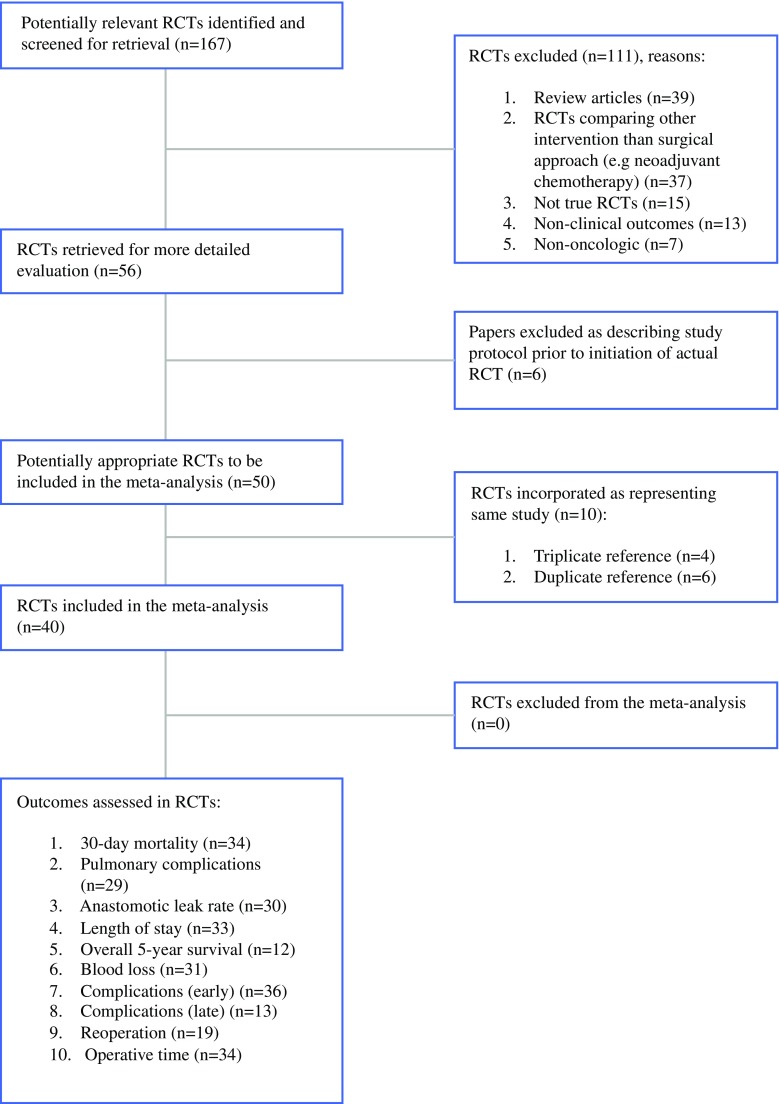
PRISMA statement flow diagram illustrating the search strategy used. *PRISMA* preferred reporting items for systematic reviews and meta-analyses, *RCTs* randomized controlled trials

The literature search yielded 56 articles^4^,^5^,^13^–^62^ reporting results from 40 RCTs that met the inclusion criteria and were included in this study. The PRISMA statement flow diagram illustrating the search strategy is shown in Fig. 1. Of these RCTs, one compared MIS against open surgery for esophageal cancer,^14^ 11 for gastric cancer,^25^–^27^,^35^,^37^,^42^,^43^,^47^,^50^,^57^,^60^ 25 for colorectal cancer,^5^,^13^,^16^ ^22^,^28^–^30^,^34^,^36^,^38^,^40^,^44^–^46^,^48^,^49^,^51^,^52^,^58^,^61^ and 15 for rectal cancer.^4^,^15^,^23^,^24^,^31^–^33^,^39^,^41^,^53^–^56^,^59^,^62^ Overall, 11,625 patients were included from 320 centers. In total, 6210 patients were randomized to MIS; however, on completion of these RCTs, the actual number of patients who underwent MIS was 5423 as 787 patients had crossed over to the open surgery arm, resulting in a mean crossover rate of 12.6% (range 0–45%). In other words, one in eight patients randomized to MIS underwent open surgery, although they were analyzed as part of the MIS group as intention-to-treat analysis was employed.

### Trial Characteristics and Crossover Rates

The characteristics of each RCT, including composite quality score, description of crossover (MIS to open surgery) in terms of percentage, time (i.e. pre- vs. intraoperative), and reasons given for it are presented in electronic supplementary Table S3. The percentage crossover ranged from 0 to 45% (mean 12.6%, standard deviation 10.6%) and primarily related to unplanned intraoperative open conversion. Electronic supplementary Table S4 illustrates further information, including patient demographics, cancer site and stage, morbidity, 30-day mortality, and overall survival (1–5 years, including median survival for each approach) for each RCT.

### The Effect of Pretrial Surgeon Volume, Composite Randomized Controlled Trial Quality Score, Site of Tumor, and American Society of Anesthesiologists Score on Crossover

The pretrial surgeon volume (*β* = − 2.344; *p* = 0.037), composite RCT quality score (*β* = − 7.594; *p* *=* 0.014), and site of tumor (*β* = − 12.031; *p* *=* 0.021, favoring lower over upper gastrointestinal tumors) showed an inverse relationship with crossover, while ASA score showed a positive relationship with crossover (*β* = + 0.895; *p* *=* 0.050) [Table 1]. When performing a subanalysis of lower gastrointestinal tumors into colonic and rectal sites, mean crossover rate was found to be higher in the former (11.3 vs. 10.7%), although the difference did not reach statistical significance (*p* > 0.05). This finding is likely to be a result of differences in comorbidity between patients included in these studies, with the rectal cancer studies consisting of patients with lower mean ASA score (i.e. less comorbid population compared with the colonic cancer studies).

**TABLE 1:** Results of univariate and multivariate linear regression illustrating the relationship between different factors and crossover (MIS to open surgery)

Univariate linear regression model	Coefficients		*p*-Value^a^	95% CI for B		Collinearity statistics
	B	SE		Lower bound	Upper bound	Variance inflation factor
No. of centers	− 0.279	0.138	0.136	− 0.717	0.159	6.436
Pretrial surgeon volume	3.844	1.133	**0.047**	0.546	12.142	19.073
Sample size	0.010	0.004	0.070	− 0.002	0.023	4.075
Composite quality score	− 9.992	1.733	**0.010**	− 15.505	− 4.478	3.235
Year of publication	0.219	0.121	0.367	− 0.165	0.603	28.403
Site of tumor (upper vs. lower GI cancer)	− 23.031	6.224	**0.034**	− 42.837	− 3.224	9.073
ASA score (3 + 4 versus 1 + 2)	0.795	0.270	**0.049**	0.013	1.653	12.534
Disease stage (III + IV versus I + II)	0.239	0.070	**0.042**	0.015	0.462	11.279
Dependent variable: MIS to open crossover (%)						

Multivariate linear regression model	Coefficients		*p*-Value^a^	95% CI for B	
	B	SE		Lower bound	Upper bound
Pretrial surgeon volume	− 2.344	0.323	**0.037**	0.546	1.142
Composite quality score	− 7.594	1.984	**0.014**	− 15.505	− 4.478
Site of tumor (upper vs. lower GI cancer)	− 12.031	3.387	**0.021**	− 22.376	− 2.340
ASA score (3 + 4 vs. 1 + 2)	0.895	0.279	**0.050**	0.001	1.253
Disease stage (III + IV vs. I + II)	0.239	1.070	0.122	− 0.789	1.462
Dependent variable: MIS to open crossover (%)					

^a^ Boldface denotes statistical significance

*SE* standard error, *CI* confidence interval, *MIS* minimally invasive surgery, *GI* gastrointestinal, *ASA* American Society of Anesthesiologists

### The Effect of Crossover on 30-Day Mortality and 30-Day Complications

Meta-regression analysis revealed a statistically significant positive correlation between crossover and 30-day mortality (*β* = + 0.125; *p* *=* 0.033), anastomotic leak rate (*β* = + 0.550; *p* *=* 0.004), and 30-day complications (*β* = + 1.255; *p* *=* 0.001), based on intention-to-treat analysis (Table 2). No statistically significant correlations were found between crossover and pulmonary complications (*β* = + 0.223; *p* *=* 0.728) or length of hospital stay (*β* = − 1.718; *p* *=* 0.939). It was not possible to perform the regression for the effect of crossover on overall 5-year survival due to insufficient data reported.

**Table 2:** Results of univariate and multivariate weighted linear regression illustrating the relationship between crossover (MIS to open surgery) and surgical endpoints

Univariate weighted linear regression			Multivariate weighted linear regression		
Factor	*β* coefficient	*p*-Value^a^	*β* coefficient	95% CI	*p*-Value^a^
*30-Day mortality*					
MIS to open crossover	0.028	**0.001**	0.125	0.012–0.238	**0.033**
Composite quality score	0.317	0.452	0.317	− 0.494 to 1.128	0.404
Number of centers	− 0.035	0.763	− 0.035	− 0.130 to 0.061	0.439
Pretrial surgeon volume	0.005	0.356	0.005	− 0.018 to 0.028	0.640
Sample size	0.001	0.645	0.001	− 0.004 to 0.003	0.907
ASA score	0.021	**0.049**	0.019	0.001–0.321	**0.049**
*Anastomotic leak*					
MIS to open crossover	0.467	**0.003**	0.550	0.259–0.841	**0.004**
Composite quality score	0.748	0.847	0.605	− 1.037 to 2.248	0.402
Number of centers	− 0.076	0.456	− 0.097	− 0.298 to 0.108	0.283
Pretrial surgeon volume	− 0.287	0.245	− 0.009	− 0.053 to 0.034	0.613
Sample size	− 0.038	0.837	− 0.005	− 0.013 to 0.003	0.181
ASA score	0.056	**0.050**	0.089	− 0.023 to 0.009	0.161
*Pulmonary complications*					
MIS to open crossover	0.223	0.874	0.223	− 1.754 to 2.201	0.728
Composite quality score	− 2.033	0.710	− 2.033	− 20.16 to 16.09	0.745
Number of centers	0.247	0.653	0.247	− 1.251 to 1.745	0.636
Pretrial surgeon volume	− 0.010	0.873	− 0.010	− 0.201 to 0.180	0.873
Sample size	− 0.012	0.541	− 0.012	− 0.049 to 0.025	0.368
ASA score	0.142	0.123	− 0.098	− 0.187 to 0.289	0.256
*Length of stay*					
MIS to open crossover	− 2.234	0.939	− 1.718	− 47.92 to 44.49	0.939
Composite quality score	− 7.276	0.977	− 4.899	− 350.2 to 340.3	0.977
Number of centers	− 0.962	0.965	− 0.661	− 32.02 to 30.69	0.965
Pretrial surgeon volume	− 2.882	0.685	− 1.800	− 10.985 to 7.386	0.685
Sample size	− 0.198	0.952	− 0.038	− 1.324 to 1.249	0.952
ASA score	− 0.763	0.456	− 0.305	− 0.013 to 0.403	0.381
*Early (30-day) complications*					
MIS to open crossover	1.255	**0.001**	1.255	0.929–4.412	**0.001**
Composite quality score	0.372	0.085	0.372	− 0.685 to 8.711	0.085
Number of centers	− 0.158	0.586	− 0.158	− 0.031 to 0.019	0.586
Pretrial surgeon volume	0.119	0.613	0.119	− 0.106 to 0.170	0.613
Sample size	− 0.174	0.586	− 0.174	− 0.031 to 0.019	0.586
ASA score	0.987	**0.009**	1.008	0.328–3.910	**0.039**

^a^Boldface denotes statistical significance

*CI* confidence interval, *MIS* minimally invasive surgery, *ASA* American Society of Anesthesiologists

## Discussion

The findings of this study demonstrate that crossover in surgical oncology RCTs is common, affecting one in eight patients. Moreover, its incidence was shown to reduce with increasing surgeon experience and decreasing patient comorbidity (as indicated by pretrial volume and ASA score, respectively). Importantly, the clinical consequences of this crossover within RCTs included increases in 30-day mortality, anastomotic leak rate, and 30-day complications demonstrated by meta-regression.

It thus becomes apparent that in the presence of crossover, an intention-to-treat analysis may underestimate the underlying mortality benefit associated with MIS, i.e. the benefit that would have been observed had crossover not occurred due to partial homogenization of the study groups. Similarly, the anastomotic leak rate may be overestimated in the MIS group. In other words, in the presence of crossover, a simple intention-to-treat analysis may result in bias that will be equal to the difference between the underlying mortality difference and the observed one (in the presence of crossover); however, the extent of this bias remains unknown (as the underlying mortality difference is not directly observed). Based on our analytical methodology, the interpretation of results offers an association between outcomes with counterfactual treatment effects, although this is not a measure of causation.^63^ What is clear though is that as long as there is a difference between MIS and open surgery, some bias will inevitably exist as a direct result of crossover.^64^

Hence, in the presence of crossover, any clinical, cost effectiveness, and economic evaluation relying on intention-to-treat analysis is prone to generate inaccurate results, which may in turn lead to inappropriate resource allocation.^64^ This is of special importance in the current healthcare climate characterized by severe financial restraints and competing national priorities for investment.^1^,^65^

Crossover represents a particularly challenging problem for bodies such as Health Technology Assessment (HTA) and the National Institute for Health and Care Excellence (NICE) in the UK, and the Agency for Healthcare Research and Quality (AHRQ) in the US, which rely on the findings of RCTs to form the basis of health policy.^66^ Surgical oncology is particularly prone to crossover due to adverse events or technical challenges experienced with the MIS techniques. Moreover, surgical RCTs in general have additional limitations.^1^ For example, when evaluating a novel medical device or surgical technique, assessment of endpoints is not commonly blind and may be affected by assessor bias added to crossover.^67^^–^ 70

Thus, it becomes apparent that there is a need to pre-emptively address factors that influence crossover. To optimize surgical oncology RCT validity, prevention strategies need to be employed to minimize and reduce the effects of crossover. Of course, crossover cannot be completely abolished for several reasons, including clinical (e.g. need for open conversion due to technical reasons or adverse event), logistic (e.g. equipment malfunction or no available surgeon competent in MIS technique for oncologic purposes), and ethical (e.g. post-randomization patient choice).^1^

In terms of prevention strategies, the current study shows that credentialing surgeons and having a minimum procedural volume threshold to enter the study reduces the degree of crossover from MIS to open surgery. Prior studies have highlighted the presence of a proficiency-gain curve with the introduction of MIS, which can substantially influence rates of conversion (crossover) and clinical endpoints, including mortality at a national level.^71^ The length of this proficiency-gain curve must be accurately defined in order to provide appropriately validated procedural volume thresholds for surgeon inclusion within RCTs. This will ensure that surgeons within their MIS proficiency-gain curve are excluded from the study.

Another prevention strategy may involve alternative processes of surgeon credentialing with video assessment and data monitoring in a pretrial phase prior to inclusion within the RCT, in a similar manner to a driving test before embarking on driving independently. COLOR-III is a good example, given the relative novelty of the procedure (transanal total mesorectal excision) and the presumption that many surgeons will currently be within their proficiency-gain curve, a pretrial phase with careful surgeon credentialing has been designed to ensure surgeons entering the RCT are beyond their period of gaining proficiency.^72^

Moreover, given its independence as a predictor of crossover, as well as its potential effect on complications, it may be advisable to include low comorbidity (ASA score) patients in RCTs only when initially comparing novel MIS techniques with open surgery. This strategy aims to further minimize crossover, promote patient safety (by reducing complications), and optimize trial validity. Depending on initial RCT findings, inclusion criteria can then be expanded in subsequent RCTs to include higher comorbidity patients.

As crossover is not random, simple methods to address it, such as excluding or censoring patients, will only lead to further bias. This is especially true when crossover is associated with prognosis, the primary endpoint in the majority of surgical oncology RCTs. Although more complex statistical methods have been developed to account for the crossover effect, it is important to appreciate that no method is superior, each suffering its own limitations.^64^

A more pragmatic approach could involve reporting endpoints in three rather than two groups, i.e. the (completed) MIS, open, and converted (crossover) trial arms in addition to the traditional intention-to-treat analysis. Only three RCTs reported endpoints in this way.^34^,^38^,^46^ This type of analysis will also allow the evaluation of factors associated with crossover, and thus try to predict which patients would not constitute good candidates for MIS due to the high risk of conversion and thus complications. Interestingly, one RCT reported endpoints using both intention-to-treat analysis and actual treatment groups separately for the same patient cohort.^19^ This is the only RCT that used both statistical methods, and illustrates the significantly worse outcomes associated with the crossover group over both the open and MIS groups.

It is important to acknowledge the limitations of this study. First, this was a study-based (as opposed to individual patient data) meta-regression analysis, and hence individual clinicopathological parameters were not included. However, in the context of RCTs, these parameters are carefully controlled for in the study design. Second, assessing the role of crossover itself is not directly quantifiable (i.e. how outcomes would differ had there been no crossover) based on the current dataset.^19^

## Conclusions

There are two key findings from this study. First, pretrial surgeon volume and patient ASA score are the two modifiable factors associated with crossover in surgical oncology RCTs. Second, the presence of crossover is associated with an increase in 30-day mortality, anastomotic leak rate, and early complications within the MIS group based on intention-to-treat-analysis. However, the association reported here is not a measure of causation. Credentialing surgeons by procedural volume is an important method of ensuring surgeons have completed their proficiency-gain curve and thus reduce the incidence of crossover, as is including low comorbidity patients only when initially comparing novel MIS techniques with open surgery. Future RCTs must develop and implement strategies, including pretrial phases and surgeon credentialing by volume or video assessment, in addition to excluding high-comorbidity patients during initial trials to reduce the incidence of crossover and thus maintain randomized homogenous groups to adequately test the study hypotheses.

## Electronic supplementary material

Below is the link to the electronic supplementary material.

Supplementary material 1 (DOCX 274 kb)
