# Supplementary material for: Induced Bias Due to Crossover Within Randomized Controlled Trials in Surgical Oncology: A Meta-regression Analysis of Minimally Invasive versus Open Surgery for the Treatment of Gastrointestinal Cancer
Source: Ann Surg Oncol. 2017 Nov 6;25(1):221–30. doi: 10.1245/s10434-017-6210-y (PMC5740197; doi:10.1245/s10434-017-6210-y)
Supplement: Supplementary file 1 — Supplementary material 1 (DOCX 274 kb) [file 10434_2017_6210_MOESM1_ESM.docx]

**Online Supplement**

| **Composite quality score of a Randomized Controlled Trial (RCT) calculation** | | |
| --- | --- | --- |
| **Jadad score** | Was the study described as randomized? | +1 / 0 |
|  | The method of randomization was described in the article, and that method was appropriate | +1 / 0 |
|  | Was the study described as double blind? | +1 / 0 |
|  | The method of blinding was described, and it was appropriate | +1 / 0 |
|  | Was there a description of withdrawals and dropouts? | +1 / 0 |
|  | The method of randomization was described but was inappropriate | -1 / 0 |
|  | The method of blinding was described but was inappropriate | -1 / 0 |
|  | Total (Range) | 0 - 5 |
| **Risk-of-bias score** | Adequate sequence generation | +1 / -1 / 0 |
|  | Allocation concealment | +1 / -1 / 0 |
|  | Blinding | +1 / -1 / 0 |
|  | Incomplete outcome data assessment | +1 / -1 / 0 |
|  | Freedom from selective reporting | +1 / -1 / 0 |
|  | Freedom from other bias | +1 / -1 / 0 |
|  | Total (Range) | 0 - 6 |
| **Composite quality score** | Sum of Jadad score + Risk-of-bias score | 0 – 11  ( > 3, threshold of rigor) |

**Table S1.** Calculation of the composite quality score for randomised controlled trials (RCTs).

**MOOSE Checklist for Meta-analyses of Observational Studies**

| **Item No** | **Recommendation** | **Reported on Page No** |
| --- | --- | --- |
| Reporting of background should include | | |
| 1 | Problem definition | 5 |
| 2 | Hypothesis statement | 5 |
| 3 | Description of study outcome(s) | 7 |
| 4 | Type of exposure or intervention used | 6 |
| 5 | Type of study designs used | 6 |
| 6 | Study population | 6 |
| Reporting of search strategy should include | | |
| 7 | Qualifications of searchers (eg, librarians and investigators) | 7 |
| 8 | Search strategy, including time period included in the synthesis and key words | 6 |
| 9 | Effort to include all available studies, including contact with authors | 6 |
| 10 | Databases and registries searched | 6 |
| 11 | Search software used, name and version, including special features used (eg, explosion) | 6 |
| 12 | Use of hand searching (eg, reference lists of obtained articles) | 6 |
| 13 | List of citations located and those excluded, including justification | 6 |
| 14 | Method of addressing articles published in languages other than English | 6 |
| 15 | Method of handling abstracts and unpublished studies | 6 |
| 16 | Description of any contact with authors | N/A |
| Reporting of methods should include | | |
| 17 | Description of relevance or appropriateness of studies assembled for assessing the hypothesis to be tested | 9 |
| 18 | Rationale for the selection and coding of data (eg, sound clinical principles or convenience) | 9,10 |
| 19 | Documentation of how data were classified and coded (eg, multiple raters, blinding and interrater reliability) | 9,10 |
| 20 | Assessment of confounding (eg, comparability of cases and controls in studies where appropriate) | 7 |
| 21 | Assessment of study quality, including blinding of quality assessors, stratification or regression on possible predictors of study results | 7 |
| 22 | Assessment of heterogeneity | 7 |
| 23 | Description of statistical methods (eg, complete description of fixed or random effects models, justification of whether the chosen models account for predictors of study results, dose-response models, or cumulative meta-analysis) in sufficient detail to be replicated | 7,8 |
| 24 | Provision of appropriate tables and graphics | 18-21 |
| Reporting of results should include | | |
| 25 | Graphic summarizing individual study estimates and overall estimate | 18-21 |
| 26 | Table giving descriptive information for each study included | 18-21 |
| 27 | Results of sensitivity testing (eg, subgroup analysis) | 18-21 |
| 28 | Indication of statistical uncertainty of findings | 18-21 |

| **Item No** | **Recommendation** | **Reported on Page No** |
| --- | --- | --- |
| Reporting of discussion should include | | |
| 29 | Quantitative assessment of bias (eg, publication bias) | 7 |
| 30 | Justification for exclusion (eg, exclusion of non-English language citations) | 6 |
| 31 | Assessment of quality of included studies | 7 |
| Reporting of conclusions should include | | |
| 32 | Consideration of alternative explanations for observed results | 11-15 |
| 33 | Generalization of the conclusions (ie, appropriate for the data presented and within the domain of the literature review) | 11-15 |
| 34 | Guidelines for future research | 15 |
| 35 | Disclosure of funding source | 16 |

**Table S2.** MOOSE checklist for Meta-analysis of Observational Studies. MOOSE = Meta-analysis Of Observational Studies in Epidemiology

| **Author** | **Year** | **Country** | **No. of centers** | **Intervention** | **CQS** | **Minimum pre-trial surgeon volume (MIS)** | **% crossover (MIS to open surgery)** | **Pre-operative crossover (%)** | **Reasons given for pre-operative crossover** | **Intra-operative crossover (%)** | **Reasons given for intra-operative crossover** |
| --- | --- | --- | --- | --- | --- | --- | --- | --- | --- | --- | --- |
| Bierre SS *et al.*^14^ | 2012 | Netherlands | 5 | Minimally invasive vs. open (transthoracic) esophagectomy for esophageal cancer | 5 | 10 | 17 | 3.4 | Increase in WHO-ECOG score to 3 | 13.6 | Technical difficulties requiring open conversion |
| Kitano S *et al.* ^47^ | 2002 | Japan | 1 | Laparoscopic vs. open distal gastrectomy for early gastric cancer | 4 | Not quantified | 0 | 0 | Not applicable | 0 | Not applicable |
| Lee JH *et al.* ^50^ | 2004 | South Korea | 1 | Laparoscopic vs. open distal gastrectomy for early gastric cancer | 4 | Not quantified | 0 | 0 | Not applicable | 0 | Not applicable |
| Sakuramoto S *et al.* ^57^ | 2013 | Japan | 1 | Laparoscopic vs. open distal gastrectomy for early gastric cancer | 6 | At least 100 laparoscopic and 500 open gastrectomies | 0 | 0 | Not applicable | 0 | Not applicable |
| Tagikuchi S *et al.* ^60^ | 2013 | Japan | 1 | Laparoscopic vs. open distal gastrectomy | 4 | More than 100 laparoscopic and 100 open distal gastrectomies | 0 | 0 | Not applicable | 0 | Not applicable |
| Cai J *et al.* ^35^ | 2011 | China | 1 | Laparoscopic vs. open D2 radical gastrectomy for advanced gastric cancer | 5 | Not quantified | 3.3 | 0 | Not applicable | 3.3 | One patient had an overlarge tumor and the other could not bear the pneumoperitoneum |
| Cui M *et al.* ^37^ | 2015 | China | 1 | Laparoscopic vs. open gastrectomy for gastric cancer | 5 | More than 150 laparoscopic gastrectomies | 7.5 | 0 | Not applicable | 7.5 | Not clarified |
| Hayashi H *et al.* ^42^ | 2005 | Japan | 1 | Laparoscopic vs. open distal gastrectomy for early gastric cancer | 4 | Not quantified | 0 | 0 | Not applicable | 0 | Not applicable |
| Huscher CGS *et al.* ^43^ | 2005 | Italy | 1 | Laparoscopic vs. open subtotal gastrectomy for gastric cancer | 4 | Not quantified | Not stated | Unknown | Unknown | Unknown | Unknown |
| Kim YW *et al.*^27^ | 2013 | South Korea | 1 | Laparoscopic vs. open gastrectomy for early gastric cancer | 6 | Not quantified | 1.2 | 0 | Not applicable | 1.2 | Intraoperative bleeding necessitating open conversion |
| Kim YW *et al.*^26^ | 2008 | South Korea | 1 | Laparoscopic vs. open gastrectomy for early gastric cancer | 6 | Not quantified | 1.2 | 0 | Not applicable | 1.2 | Intraoperative bleeding necessitating open conversion |
| Kim HH *et al.*^25^ | 2010 | South Korea | 10 | Laparoscopic vs. open gastrectomy for gastric cancer | 7 | 50 (plus 80 cases in their institution each year) | 0 | 0 | Not applicable | 0 | N/A |
| Lacy AM *et al.* ^49^ | 2002 | Spain | 1 | Laparoscopic vs. open colectomy for colonic cancer | 5 | Not quantified | 11 | 0 | Not applicable | 11 | Suspected tumor invasion of adjacent organs |
| Chung CC *et al.* ^36^ | 2007 | China | 1 | Laparoscopic vs. open right hemicolectomy for right colonic cancer | 5 | Not quantified | 7 | 0 | Not applicable | 7 | Adhesions in two patients and ureteric injury in third |
| Braga M *et al.* ^33^ | 2007 | Italy | 1 | Laparoscopic vs. open rectal resection for rectal cancer | 4 | Not quantified | 7.2 | 0 | Not applicable | 7.2 | Narrow pelvis, difficulty in transecting the distal rectum |
| Ng SSM *et al.* ^55^ | 2009 | China | 1 | Laparoscopic vs. open anterior resection for upper rectal cancer | 4 | Not quantified | 30 | 0 | Not applicable | 30 | Local tumor invasion, bulky tumor, narrow pelvis, dilated small bowel obstructing view, dense adhesions, bleeding, rectal perforation, and anastomotic failure |
| Gonzalez IA *et al.* ^32^ | 2006 | Spain | 1 | Laparoscopic vs. open surgery for rectal cancer | 4 | Not quantified | 10 | 0 | Not applicable | 10 | Difficult access due to obesity, large tumor |
| Li JCM *et al.* ^51^ | 2012 | China | 1 | Laparoscopic vs. open right hemicolectomy for right colonic cancer | 4 | Not quantified | 16 | 0 | Not applicable | 16 | Uncontrolled bleeding, surgical emphysema, failed tumor localisation, extensive adhesions, and bulky tumor |
| Winslow ER *et al.* ^61^ | 2002 | USA | 1 | Laparoscopic vs. open colectomy for colonic cancer | 4 | Not quantified | 15 | 0 | Not applicable | 15 | Not clarified |
| Zhou ZG *et al.* ^62^ | 2004 | China | 1 | Laparoscopic vs. open total mesorectal excision with anal sphincter preservation for low rectal cancer | 3 | Not quantified | Not stated | Unknown | Unknown | Unknown | Unknown |
| Fuji S *et al.* ^40^ | 2014 | Japan | 1 | Laparoscopic vs. open resection in elderly colorectal cancer patients | 6 | Not quantified | 3 | 1 | Patient’s desire | 2 | Uncontrollable bleeding and excision of a peritoneal metastasis in a patient with transverse colon cancer |
| Schwenk W *et al.* ^58^ | 1998 | Germany | 1 | Laparoscopic vs. open colectomy for colorectal cancer | 4 | Not quantified | 13 | 0 | Not applicable | 13 | Not clarified |
| Curet MJ *et al.* ^38^ | 2000 | USA | 1 | Laparoscopic vs. open colectomy for colonic cancer | 4 | Not quantified | 28 | 0 | Not applicable | 28 | Tumor fixation to adjacent tissues or technical reasons |
| Lacy AM *et al.* ^48^ | 1998 | Spain | 1 | Laparoscopic vs. open colectomy for colonic cancer | 3 | Not quantified | 13 | 0 | Not applicable | 13 | Tumor invading adjacent organs |
| Liang JT *et al.* ^52^ | 2007 | Taiwan | 1 | Laparoscopic vs. open colectomy for colonic cancer | 6 | Not quantified | 2.8 | 0 | Not applicable | 2.8 | One poor visualisation, one massive bleeding, and two incomplete mobilisation due to adhesions and suspected local tumor invasion |
| Ng SSM *et al.* ^56^ | 2008 | China | 1 | Laparoscopic vs. open abdominal-perineal resection for low rectal cancer | 4 | Not quantified | 9.8 | 0 | Not applicable | 9.8 | Failure to identify the left ureter, bleeding, unexpected pelvic side wall invasion, and bulky tumor |
| Buchanan GN *et al.* ^34^ | 2008 | UK | 1 | Laparoscopic vs. open resection for colorectal cancer | 4 | More than 130 laparoscopic operations for benign colorectal disease | 17 | 0 | Not applicable | 17 | Reasons given include technical, oncological, adhesions from previous surgery, obesity, anesthetic reasons, hemorrhage, to achieve tumor clearance, and ureteric injury |
| Kaiser AM *et al.* ^46^ | 2004 | USA | 1 | Laparoscopic vs. open surgery for colonic cancer | 3 | Not quantified | 45 | 0 | Not applicable | 45 | Reasons given include stage IV disease, immobile colon, adhesions, bleeding, difficulty in identifying ureter, need for simultaneous cholecystectomy |
| Liang X *et al.* ^53^ | 2011 | China | 1 | Laparoscopic vs. open surgery for rectal cancer | 4 | Not quantified | 0.6 | 0 | Not applicable | 0.6 | Bowel adhesion |
| Lujan J *et al.* ^54^ | 2009 | Spain | 1 | Laparoscopic vs. open surgery for rectal cancer | 5 | Not quantified | 7.9 | 0 | Not applicable | 7.9 | Inability to complete distal section of total mesorectal excision, difficulty in mobilizing the splenic flexure, bleeding of the presacral bed, an ectopic kidney, ischemia of the descending colon, and ureteral injury |
| Gong J *et al.* ^41^ | 2012 | China | 1 | Laparoscopic vs. open surgery for rectal cancer | 3 | More than 100 laparoscopic and open colorectal surgeries annually | 3 | 0 | Not applicable | 3 | Ureteric injury and adhesions |
| Hewett PJ *et al.*^20^ | 2008 | Australia | 31 | Laparoscopic vs. open colectomy for colonic cancer | 5 | Not quantified | 18 | 3.1 | Randomized to laparoscopic arm but operated open in error | 14.9 | Intraoperative factors, multiple quoted such as inability to visualize critical structures, advanced disease, inability to mobilize the colon etc. |
| Bagshaw PF *et al.*^13^ | 2012 | Australia | 31 | Laparoscopic vs. open colectomy for colonic cancer | 5 | Not quantified | 18 | 3.1 | Randomised to laparoscopic arm but operated open in error | 14.9 | Intraoperative factors, multiple quoted such as inability to visualize critical structures, advanced disease, inability to mobilize the colon etc. |
| Guillou PJ *et al.*^19^ | 2005 | UK | 27 | Laparoscopic vs. open colectomy for colorectal cancer | 4 | 20 | 29 | 0 | Not applicable | 29 | Difficulty to remove tumor, uncertainty of tumor clearance, obesity |
| Jayne DG *et al.*^21^ | 2007 | UK | 27 | Laparoscopic vs. open colectomy for colorectal cancer | 4 | 20 | 29 | 0 | Not applicable | 29 | Difficulty to remove tumor, uncertainty of tumor clearance, obesity |
| Jayne DG *et al.*^22^ | 2010 | UK | 27 | Laparoscopic vs. open colectomy for colorectal cancer | 4 | 20 | 29 | 0 | Not applicable | 29 | Difficulty to remove tumor, uncertainty of tumor clearance, obesity |
| Veldkamp R *et al.*^5^ | 2005 | Canada | 29 | Laparoscopic vs. open colectomy for colonic cancer | 5 | 20 | 19 | 2 | Malfunction of laparoscopic equipment or laparoscopic team absent | 17 | Various reasons incl. fixation or invasion of adjacent structures by tumor, size of tumor, extensive adhesions, inability to localize tumor, bleeding etc. |
| Bonjer HJ^17^ | 2009 | Canada | 29 | Laparoscopic vs. open colectomy for colonic cancer | 5 | 20 | 19 | 2 | Malfunction of laparoscopic equipment or laparoscopic team absent | 17 | Various reasons incl. fixation or invasion of adjacent structures by tumor, size of tumor, extensive adhesions, inability to localize tumor, bleeding etc. |
| Bonjer HJ *et al.*^15^ | 2015 | Netherlands | 30 | Laparoscopic vs. open surgery for rectal cancer | 5 | Not quantified | 17 | 1 | One due to pulmonary function, 5 because no laparoscopic surgeon was available, and one for unknown reason | 16 | Not clarified |
| Van der Pas MH *et al.*^4^ | 2013 | Netherlands | 30 | Laparoscopic vs. open surgery for rectal cancer | 5 | Not quantified | 17 | 1 | One due to pulmonary function, 5 because no laparoscopic surgeon was available, and one for an unknown reason | 16 | Not clarified |
| Andersson J *et al.* ^31^ | 2014 | Sweden | 12 | Laparoscopic vs. open surgery for rectal cancer | 5 | Not quantified | 0 | 0 | Not applicable | 0 | Not applicable |
| Janson M *et al.* ^45^ | 2007 | Sweden | 7 | Laparoscopic vs. open surgery for colon cancer | 5 | Not quantified | 18 | 0 | Not applicable | 18 | Not clarified |
| Janson M et al. ^44^ | 2004 | Sweden | 10 | Laparoscopic vs. open surgery for colon cancer | 5 | 20 | 14 | 0 | Not applicable | 14 | Not clarified |
| Jeong SY *et al.*^23^ | 2014 | South Korea | 3 | Laparoscopic vs. open surgery for rectal cancer | 6 | 91 | 1 | 0 | Not applicable | 1 | See below |
| Kang SB *et al.*^24^ | 2010 | South Korea | 3 | Laparoscopic vs. open surgery for rectal cancer | 6 | 91 | 1 | 0 | Not applicable | 1 | Difficult dissection in narrow pelvis and intraoperative bleeding |
| Weeks JC *et al.*^29^ | 2002 | USA | 37 | Laparoscopic vs. open colectomy for colonic cancer | 4 | 20 | 26 | 0 | Not applicable | 26 | Advanced disease, positive margins, inability to adequately visualize critical structures, inability to mobilize colon, adhesions, intraoperative complications, associated complicating disease, other |
| Nelson H *et al.*^16^ | 2004 | USA | 48 | Laparoscopic vs. open colectomy for colonic cancer | 4 | 20 | 21 | 0 | Not applicable | 21 | Advanced disease, complicating disease, inadequate margins of resection, no visualization of critical structures, unable to mobilize colon, adhesions, intraoperative complications, other |
| Flesman J *et al.*^18^ | 2007 | USA | 48 | Laparoscopic vs. open colectomy for colonic cancer | 4 | 20 | 21 | 0 | Not applicable | 21 | Advanced disease, complicating disease, inadequate margins of resection, no visualization of critical structures, unable to mobilize colon, adhesions, intraoperative complications, other |
| Yamamoto S *et al.*^30^ | 2014 | Japan | 30 | Laparoscopic vs. open D3 dissection for stage II/III colonic cancer | 6 | 30 MIS and 30 open | 6 | 0.5 | Patient refusal, hepatic metastases, subileus, synchronous multiple cancers | 5.5 | Indicated conversion: tumor invasion to adjacent structures, peritoneal dissemination, synchronous tumors, synchronous hepatic metastases and technical conversion: intraoperative bleeding, organ injury, inability to mobilize the colon, inability to visualize, difficulty in rectal transection, and complicated conversion: problems with anesthetic management |
| Neudecker J *et al.*^28^ | 2009 | Germany | 20 | Laparoscopic vs. open colectomy for colorectal cancer | 6 | 20 | 11 | 0 | Not applicable | 11 | Bleeding, severe adhesions, technical problems, uncertain anatomy, tumor size, etc. |
| Flesman J *et al.* ^39^ | 2015 | USA | 35 | Laparoscopic vs. open resection for rectal cancer | 5 | At least 20 MIS and 20 open proctectomies | 11 | 0 | Not applicable | 11 | Unable to complete dissection safely, locally advanced disease discovered at surgery, complications during surgery, unable to complete anastomosis safely, adhesions |
| Stevenson A *et al.* ^59^ | 2015 | Australia | 24 | Laparoscopic vs. open surgery for rectal cancer | 6 | At least 100 MIS colon resections and 30 MIS rectal resections | 9 | 0 | Not applicable | 9 | pT4 tumor for 2 patients, reasons unclear for remainder of converted patients |

**Table S3.** Included RCTs with demographic characteristics, composite quality scores, and description of crossover (MIS to open surgery) in terms of percentage, timing, and reasons given for it. RCT = randomized controlled trial; CQS = composite quality score; MIS = minimally invasive surgery

| **Author** | **Time Period** | **Cancer site** | **Disease stage** | | **Median Follow-up Period (Months) (Range)** | | **Patient Number** | | **Median Age**  **(Range/SD)** | | **Male/Female ratio** | | **Morbidity** | | **30-day Mortality** | | **Overall survival** | | | | | | | | | |
| --- | --- | --- | --- | --- | --- | --- | --- | --- | --- | --- | --- | --- | --- | --- | --- | --- | --- | --- | --- | --- | --- | --- | --- | --- | --- | --- |
|  |  |  |  |  |  |  |  |  |  |  |  |  |  |  |  |  | **1-year** | | **2-year** | | **3-year** | | **5-year** | | **Median (Months)** | |
|  |  |  | **MIS** | **OS** | **MIS** | **OS** | **MIS** | **OS** | **MIS** | **OS** | **MIS** | **OS** | **MIS** | **OS** | **MIS** | **OS** | **MIS** | **OS** | **MIS** | **OS** | **MIS** | **OS** | **MIS** | **OS** | **MIS** | **OS** |
| Bierre SS *et al.*^14^ | 2009-2011 | Esophagus | I(4), IIa(17), IIb(9), III(11), IV(4) | I(4), IIa(16), IIb(6), III(14), IV(5) | NR | NR | 59 | 56 | 62 (34-75) | 62 (42-75) | 43/16 | 46/10 | 5(9%) | 16(29%) | 1(2%) | 0(0%) | NR | NR | NR | NR | NR | NR | NR | NR | NR | NR |
| Kitano S *et al.* ^47^ | 1998-2001 | Gastric | Ia(13), Ib(0), II(1) | Ia(14), Ib(0), II(0) | 24.3+9.6 | 18.8+12.4 | 14 | 14 | 63.2(3.0) | 60.1(3.4) | 9/5 | 8/6 | 2(14%) | 4(28%) | 0(0%) | 0(0%) | NR | NR | NR | NR | NR | NR | NR | NR | NR | NR |
| Lee JH *et al.* ^50^ | 2001-2003 | Gastric | Ia(21), Ib(2), IIIa(1) | Ia(22), Ib(1) | 14 | 14 | 24 | 23 | 56.6+11.2 | 59.5+11.6 | 11/13 | 15/8 | 3(12.5%) | 10(44%) | NR | NR | NR | NR | NR | NR | NR | NR | NR | NR | NR | NR |
| Sakuramoto S *et al.* ^57^ | 2005-2008 | Gastric | Ia(26), Ib(3), II(1),IIIa(1) | Ia(28), Ib(4), II(0), IIIa(0) | 69 (55-89) (cumulative) | 69 (55-89) (cumulative) | 31 | 32 | 58+9.6 | 61+7.6 | 17/14 | 25/7 | 1(3.2%) | 5(15.6%) | 0(0%) | 0(0%) | NR | NR | NR | NR | NR | NR | NR | NR | NR | NR |
| Tagikuchi S *et al.* ^60^ | 2003-2006 | Gastric | Ia(17), Ib(2), II(1) | Ia(19), Ib(0), II(1) | NR | NR | 20 | 20 | 61.5 (53.5-71) | 62.5 (55-67.5) | 12/8 | 13/7 | 0(0%) | 2(10%) | 0(0%) | 0(0%) | NR | NR | NR | NR | NR | NR | 100% | 100% | NR | NR |
| Cai J *et al.* ^35^ | 2008-2009 | Gastric | Ib(14), II(13), IIa(16), IIIb(6) | Ib(11), II(17), IIIa(15), IIIb(4) | 22.1(cumulative) | 22.1(cumulative) | 49 | 47 | 60.2+9.8 | 60.3+10.2 | 39/10 | 37/10 | 6(12.2%) | 9(19.2%) | 0(0%) | 0(0%) | NR | NR | NR | NR | 67.1% | 53.8% | NR | NR | 29.4 | 28.9 |
| Cui M *et al.* ^37^ | 2010-2012 | Gastric | Ia(10), Ib(16), IIa(17), IIb(16), IIIa(26), IIIb(23), IIIc(20) | Ia(15), Ib(18), IIa(13), IIb(26), IIIa(25), IIIb(26), IIIc(19) |  |  | 128 | 142 | 60.1+12.6 | 57.5+11.2 | 88/40 | 98/44 | 26(21.8%) | 26(19.0%) | 0(0%) | 0(0%) | NR | NR | NR | NR | NR | NR | NR | NR | NR | NR |
| Hayashi H *et al.* ^42^ | 1999-2001 | Gastric | T1(12), T2(2) | T1(14) | 39 (5-49) | 45 (34-53) | 14 | 14 | 56 (47-70) | 62 (49-75) | 9/5 | 13/1 | 5(35.7%) | 8(57.1%) | 0(0%) | 0(0%) | NR | NR | NR | NR | NR | NR | NR | NR | NR | NR |
| Huscher CGS *et al.* ^43^ | 1992-1996 | Gastric | Ia(7), Ib(6), II(4), IIIa(5), IIIb(3), IV(5) | Ia(6), Ib(3), II(5), IIIa(1), IIIb(4), IV(4) | 60 (2-88) | 55 (7-90) | 30 | 29 | 63.2+12.5 | 63.6+13.2 | 18/12 | 21/8 | 7(23.3%) | 8(27.6%) | 1(3.3%) | 2(6.7%) | NR | NR | NR | NR | NR | NR | 58.9% | 55.7% | NR | NR |
| Kim YW *et al.*^27^ | 2003-2005 | Gastric | Ia(69), Ib(11), II(2) | Ia(69), Ib(9), II(3), IIIb(1) | 74.3 (cumulative) | 74.3 (cumulative) | 82 | 82 | 56.7 (35-80) | 54.5 (28-78) | 47/35 | 52/30 | 24(29.3%) | 36(43.9%) | 0(0%) | 0(0%) | NR | NR | NR | NR | NR | NR | 97.6% | 96.3% | NR | NR |
| Kim YW *et al.*^26^ | 2003-2005 | Gastric | Ia(69), Ib(11), II(2) | Ia(69), Ib(9), II(3), IIIb(1) | NR | NR | 82 | 82 | 56.7 (35-80) | 54.5 (28-78) | 47/35 | 52/30 |  |  | 0(0%) | 4(4.9%) | NR | NR | NR | NR | NR | NR | NR | NR | NR | NR |
| Kim HH *et al.*^25^ | 2006-2007 | Gastric | NR | NR | NR | NR | 179 | 163 | 54.7 | 56.9 | 116/63 | 111/52 | 20(11.6%) | 27(15.1%) | 2(1.1%) | 0(0%) | NR | NR | NR | NR | NR | NR | NR | NR | NR | NR |
| Lacy AM *et al.* ^49^ | 1993-1998 | Colon | I(27), II(42), III(37), IV(5) | I(18), II(48), III(36), IV(6) | 44 (27-85) | 43 (27-85) | 111 | 108 | 66 (12) | 71 (11) | 56/55 | 50/58 | 12(10.9%) | 31(28.7%) | 1(1%) | 3(3%) | 90.1% | 86.1% | 74.8% | 75% | 47.8% | 44.4% | 22.5% | 20.4% | NR | NR |
| Chung CC *et al.* ^36^ | 2001-2006 | Colon | Dukes’ staging:  A(4), B(21), C(15), D(1) | Dukes’ staging: A(5), B(16), C(18), D(1) | 30 (2-60) | 28 (3-60) | 41 | 40 | 71(39-88) | 72.5(52-89) | 25/16 | 26/14 | 5(12.2%) | 9(22.5%) | 1(2.4%) | 0(0%) | NR | NR | NR | NR | NR | NR | 83% | 74% | NR | NR |
| Braga M *et al.* ^33^ | NR | Rectum | I(25), II(16), III(31), IV(11) | I(24), II(19), III(29), IV(13) | 53.6(54.2) (cumulative) | 53.6(54.2) (cumulative) | 83 | 85 | 62.8(12.6) | 65.3(10.3) | 55/28 | 64/21 | 24(28.9%) | 34(40%) | 1(1.2%) | 1(1.2%) | NR | NR | NR | NR | NR | NR | NR | NR | NR | NR |
| Ng SSM *et al.* ^55^ | 1993-2002 | Rectum | I(11), II(29), III(20), IV(16) | I(13), II(29), III(28), IV(7) | 112.5(71.1-168.3) | 108.8(69.8-168.7) | 76 | 77 | 66.5+11.9 | 65.7+12.0 | 37/39 | 48/29 | 23(30.3%) | 24(31.2%) | 2(2.6%) | 3(3.9%) | 98% | 92% | 95% | 89% | 93% | 79% | 84% | 68% | NR | NR |
| Gonzalez IA *et al.* ^32^ | 2003-2004 | Rectum | I(4), II(7), III(7), IV(2) | I(7), II(5), III(3), IV(5) | NR | NR | 20 | 20 | 66.6+12.6 | 70.7+9.2 | 11/9 | 8/12 | 7(35%) | 9(45%) | 0(0%) | 0(0%) | NR | NR | NR | NR | NR | NR | NR | NR | NR | NR |
| Li JCM *et al.* ^51^ | 1996-2005 | Colon | I(8), II(35), III(16), IV(12) | I(6), II(30), III(30), IV(8) | 104(44-156) | 99.5(44-156) | 71 | 74 | 68+11.3 | 68+13.3 | 33/38 | 32/42 | 17(23.9&) | 26(35.1%) | 0(0%) | 2(2.7%) | NR | NR | NR | NR | NR | NR | 74.2% | 75% | NR | NR |
| Winslow ER *et al.* ^61^ | 1995-2000 | Colon | NR | NR | 31.1+16.9 | 29.4+18.6 | 37 | 46 | 69.4+11.7 | 65.7+11.5 | NR | NR | NR | NR | NR | NR | NR | NR | NR | NR | NR | NR | NR | NR | NR | NR |
| Zhou ZG *et al.* ^62^ | 2001-2002 | Rectum | Dukes’ staging:  A(6), B(8), C1(35), C2(33) D(7) | Dukes’ staging:  A(5), B(10), C1(33), C2(30), D(4) | NR | NR | 82 | 89 | 45(30-81) | 44(26-85) | 43/46 | 46/36 | 11(12.4%) | 5(6.1%) | 0(0%) | 0(0%) | NR | NR | NR | NR | NR | NR | NR | NR | NR | NR |
| Fuji S *et al.* ^40^ | 2008-2012 | Colorectal | I(27), II(36), III(30), IV(2) | I(24), II(34), III(33), IV(7) | NR | NR | 100 | 100 | 79.8+3.6 | 80.1+4.2 | 50/50 | 60/40 | 23(23%) | 36(36%) | 0(0%) | 0(0%) | NR | NR | NR | NR | NR | NR | NR | NR | NR | NR |
| Schwenk W *et al.* ^58^ | 1995-1996 | Colorectal | NR | NR | NR | NR | 30 | 30 | 63.3+12.2 | 64.8+14.7 | 14/16 | 16/14 | 2(6.7%) | 8(26.7%) | NR | NR | NR | NR | NR | NR | NR | NR | NR | NR | NR | NR |
| Curet MJ *et al.* ^38^ | 1993-1995 | Colon | Dukes’ staging: A(1), B(10), C(7), D(0)  Converted: A(0), B(2), C(3), D(2) | Dukes’ staging: A(0), B(9), C(5), D(4) | 4.9 (2.5-6.3) (cumulative) | 4.9 (2.5-6.3) (cumulative) | 18 (converted: 7) | 18 | 65.6 (45-83)  (converted: 66.3 (51-76)) | 69.2 (49-82) | 11/7 (converted: 4/3) | 14/4 | 1 (converted 7) | 5 | 0 (converted 1) | 0 | NR | NR | NR | NR | NR | NR | 22.2% (converted: 14.3%) | 33.3% | NR | NR |
| Lacy AM *et al.* ^48^ | 1993-1996 | Colon | Dukes’ staging: A(4), B1(2), B2(12), C1(0), C2(8), D1(5) | Dukes’ staging: A(2), B1(3), B2(21), C1(2), C2(10), D1(2) | 21.4+11.5 (13-41) (cumulative | 21.4+11.5 (13-41) (cumulative | 31 | 40 | 71.2+9.2 | 69.7+10.2 | 24/20 | 23/24 | NR | NR | 0(0%) | 0(0%) | NR | NR | 84% | 90% | NR | NR | NR | NR | NR | NR |
| Liang JT *et al.* ^52^ | 2000-2004 | Colon | II(68), III(67) | II(64), III(70) | 40 (18-72) (cumulative) | 40 (18-72) (cumulative) | 135 | 134 | 64.4+9.4 | 64.2+12.0 | 76/59 | 71/63 | 20(14.8%) | 29(21.6%) | NR | NR | NR | NR | NR | NR | NR | NR | NR | NR | NR | NR |
| Ng SSM *et al.* ^56^ | 1994-2005 | Rectum | I(10), II(13), III(17), IV(11) | I(8), II(8), III(20), IV(12) | 87.2 (22.8-150.0) | 90.1 (27.0-145.5) | 51 | 48 | 63.7+11.8 | 63.5+12.6 | 31/20 | 30/18 | 23(45.1%) | 25(52.1%) | 1(2%) | 1(2.1%) | 76.5% | 62.5% | 74.5% | 58.3% | 66.7% | 54.2% | 47.1% | 39.6% | NR | NR |
| Buchanan GN *et al.* ^34^ | 1994-2005 | Colorectal | NR | NR |  |  | 230 | 135 | 73 (44-95) | 71 (43-93) | 120/110 | 71/64 | 71(30.9%) | 52(38.5%) | 13(5.7%) | 6(4.4%) | NR | NR | NR | NR | NR | NR | NR | NR | NR | NR |
| Kaiser AM *et al.* ^46^ | 1995-2001 | Colon | I(2), II(10), III(3), IV(0)  Converted: I(2), II(5), III(2), IV(4) | I(7), II(3), III(10), IV(0) | 35 (3-69) (cumulative) | 35 (3-69) (cumulative) | 29 | 20 | 59.0 (41-83) (converted: 60.5 (48-68)) | 60.5 (42-80) | 7/8 (converted 5/8) | 9/11 | 1(7%) (converted 4(31%)) | 4(20%) | 0(0%) | 0(0%) | NR | NR | NR | NR | NR | NR | NR | NR | NR | NR |
| Liang X *et al.* ^53^ | 2004-2008 | Rectum | T1-2N0M0(9), T3-4N0M0(72), TxN1-2M0(88) | T1-2N0M0(7), T3-4N0M0(84), TxN1-2M0(83) | 44 (1-72) (cumulative) | 44 (1-72) (cumulative) | 169 | 174 | 57.34+14.13 | 57.36+13.08 | 104/65 | 92/82 | 19(11.2%) | 21(12.1%) | 0(0%) | 0(0%) | 94% | 95.3% | 82.6% | 91.2% | 76.0% | 82.8% | NR | NR | NR | NR |
| Lujan J *et al.* ^54^ | 2002-2007 | Rectum | I(11), II(35), III(45), IV(10) | I(15), II(39), III(44), IV(5) | 32.8 (18.9) | 34.1 (20.0) | 101 | 103 | 67.8 (12.9) | 66.0 (9.9) | 62/39 | 64/39 | 34(33.7%) | 34(33.0%) | 2(1.9%) | 3(2.9%) | NR | NR | 91.1% | 89.2% | 72.1% | 75.3% | NR | NR | NR | NR |
| Gong J *et al.* ^41^ | 2008-2011 | Rectum | I(7), II(46), III(14) | I(9), II(49), III(13) | 21 (9-56) | 21 (9-56) | 67 | 71 | 58.4+13.6 | 59.6+9.4 | 1.3/1 | 1.29/1 | 4(5.97%) | 6(8.5%) | 0(0%) | 0(0%) | NR | NR | NR | NR | NR | NR | NR | NR | NR | NR |
| Hewett PJ *et al.*^20^ | 1998-2005 | Colon | I(68), II(132), III(77), IV(6) | I(71), II(107), III(100), IV(5) | NR | NR | 294 | 298 | 71.1+10.4 | 69.4+11.4 | 139/155 | 143/155 | 205(69.7%) | 276(92.6%) | 4(1.4%) | 2(0.7%) | NR | NR | NR | NR | NR | NR | NR | NR | NR | NR |
| Bagshaw PF *et al.*^13^ | 1998-2005 | Colon | I(69), II(129), III(74), IV(10) | I(71), II(107), III(95), IV(10) | 5.2 years(1-11.4) (cumulative) | 5.2 years(1-11.4) (cumulative) | 290 | 297 | 71.2+10.3 | 69.4+11.4 | NR | NR | NR | NR | NR | NR | 97.2% | 96.3% | 92.1% | 90.6% | 87.2% | 84.9% | 77.7% | 76.0% | NR | NR |
| Guillou PJ *et al.*^19^ | 1996-2002 | Colorectal | T1(26), T2(68), T3(261), T4(70) | T1(12), T2(35), T3(136), T4(33) | NR | NR | 526 | 268 | 69+11 | 69+12 | 296/230 | 145/123 | 67(13%) | 29(11%) | 21(4%) | 13(4.9%) | NR | NR | NR | NR | NR | NR | NR | NR | NR | NR |
| Jayne DG *et al.*^21^ | 1996-2002 | Colorectal | Dukes’ staging: A(88), B(182), C1(155), C2(40) | Dukes’ staging: A(44), B(99), C1(75), C2(18) | 36.8 (20.0-61.5) (cumulative) | 36.8 (20.0-61.5) (cumulative) | 526 | 268 | 69+11 | 69+12 | 296/230 | 145/123 | 67(13%) | 29(11%) | 21(4%) | 13(4.9%) | NR | NR | NR | NR | 68.4% | 66.7% | NR | NR | NR | NR |
| Jayne DG *et al.*^22^ | 1996-2002 | Colorectal | Dukes’ staging: A(88), B(182), C1(155), C2(40) | Dukes’ staging: A(44), B(99), C1(75), C2(18) | 56.3 (22.3-77.3) (cumulative) | 56.3 (22.3-77.3) (cumulative) | 526 | 268 | 69+11 | 69+12 | 296/230 | 145/123 | 67(13%) | 29(11%) | 21(4%) | 13(4.9%) | NR | NR | NR | NR | 68.4% | 66.7% | 57.9% | 58.1% | NR | NR |
| Veldkamp R *et al.*^5^ | 1997-2003 | Colon | I(129), II(218), III(181) | I(125), II(239), III(175) | NR | NR | 627 | 621 | 71 (27-92) | 71 (31-95) | 326/301 | 336/285 | 111(21%) | 110(20%) | 6(1%) | 10(2%) | NR | NR | NR | NR | NR | NR | NR | NR | NR | NR |
| Bonjer HJ^17^ | 1997-2003 | Colon | I(129), II(218), III(181) | I(125), II(239), III(175) | 53 (0.03-60) (cumulative) | 53 (0.03-60) (cumulative) | 627 | 621 | 71 (54-84) | 71 (55-83) | 277/257 | 289/253 | 111(21%) | 110(20%) | 6(1%) | 10(2%) | NR | NR | NR | NR | 81.8% | 84.2% | 73.8% | 74.2% | NR | NR |
| Bonjer HJ *et al.*^15^ | 2004-2010 | Rectum | I(231), II(180), III(233), IV(4) | I(107), II(91), III(125), IV(0) | NR | NR | 699 | 345 | 66.8+10.5 | 65.8+10.9 | 448/251 | 211/134 | NR | NR | NR | NR | NR | NR | NR | NR | 86.7% | NR | NR | NR | NR | NR |
| Van der Pas MH *et al.*^4^ | 2004-2010 | Rectum | I(201), II(209), III(257) | I(96), II(107), III(126) | NR | NR | 699 | 345 | 66.8+10.5 | 65.8+10.9 | 448/251 | 211/134 | 278(40%) | 128(37%) | 8(1%) | 6(2%) | NR | NR | NR | NR | NR | NR | NR | NR | NR | NR |
| Andersson J *et al.* ^31^ | 2004-2010 | Rectum | I(18), II(93), III(135), IV(9) | I(4), II(42), III(72), IV(3) | NR | NR | 260 | 125 | 67.4 (66.1, 68.6) | 66.6 (64.8, 68.4) | 162/98 | 77/48 | NR | NR | NR | NR | NR | NR | NR | NR | NR | NR | NR | NR | NR | NR |
| Janson M *et al.* ^45^ | 1997-2002 | Colon | I(25), II(58), III(47) | I(40), II(63), III(52) | NR | NR | 130 | 155 | 70.3 (68.2-72.5) | 70.2 (68.5-72.0) | 62/68 | 82/73 | 33(29%) | 31(28%) | NR | NR | NR | NR | NR | NR | NR | NR | NR | NR | NR | NR |
| Janson M et al. ^44^ | 1999-2002 | Colon | I(18), II(44), III(36) | I(27), II(45), III(40) | NR | NR | 98 | 112 | 71.1 (68.8, 73.4) | 69.4 (67.3, 71.5) | 51/47 | 57/55 | 21(21%) | 18(16.1%) | 0(0%) | 0(0%) | NR | NR | NR | NR | NR | NR | NR | NR | NR | NR |
| Jeong SY *et al.*^23^ | 2006-2009 | Rectum | T0-T2(95), T3-T4(75) | T0-T2(71), T3-T4(99) | NR | NR | 170 | 170 | 57.8 (11.1) | 59.1 (9.9) | 110/60 | 110/60 | NR | NR | NR | NR | 97.7% | 98.2% | 94.1% | 94.7% | 87.7% | 87.1% | 23.5% | 24.7% |  |  |
| Kang SB *et al.*^24^ | 2006-2009 | Rectum | T0-T2(95), T3-T4(75) | T0-T2(71), T3-T4(99) | NR | NR | 170 | 170 | 57.8 (11.1) | 59.1 (9.9) | 110/60 | 110/60 | 36(21.2%) | 40(23.5%) | 0(0%) | 0(0%) | NR | NR | NR | NR | NR | NR | NR | NR | NR | NR |
| Weeks JC *et al.*^29^ | 1994-1999 | Colon | I(88), II(77), III(57), IV(5) | I(69), II(78), III(62), IV(11) | NR | NR | 228 | 221 | 68.2 (28-96) | 69.4 (38-95) | 118/110 | 108/113 | NR | NR | NR | NR | NR | NR | NR | NR | NR | NR | NR | NR | NR | NR |
| Nelson H *et al.*^16^ | 1994-2001 | Colon | I(153), II(136), III(112), IV(10) | I(112), II(146), III(121), IV(16) | 4.4 (cumulative) | 4.4 (cumulative) | 435 | 428 | 70 (28-96) | 69 (29-94) | 223/212 | 208/220 | 92(21%) | 85(20%) | 2(0.5%) | 4(1%) | NR | NR | NR | NR | 86% | 85% | NR | NR | NR | NR |
| Flesman J *et al.*^18^ | 1994-2001 | Colon | I(153), II(136), III(112), IV(10) | I(112), II(146), III(121), IV(16) | NR | NR | 435 | 428 | 70 (28-96) | 69 (29-94) | 223/212 | 208/220 | 92(21%) | 85(20%) | 2(0.5%) | 4(1%) | NR | NR | NR | NR | NR | NR | 76.4% | 74.6% | NR | NR |
| Yamamoto S *et al.*^30^ | 2004-2009 | Colon | II(335), III(196), IV(2) | II(362), III(161), IV(1) | NR | NR | 533 | 524 | 64 (28-75) | 64 (33-75) | 285/248 | 309/215 | 11(2.1%) | 39(7.4%) | 0(0%) | 1(0.2%) | NR | NR | NR | NR | NR | NR | NR | NR | NR | NR |
| Neudecker J *et al.*^28^ | 1998-2004 | Colorectal | I(87), II(81), III(82) | I(63), II(85), III(74) | NR | NR | 250 | 222 | 66.8+10.1 | 66.4+11.1 | 132/118 | 116/106 | 14(5.6%) | 5(2.3%) | 3(1.2%) | 2(0.9%) | NR | NR | NR | NR | NR | NR | NR | NR | NR | NR |
| Flesman J *et al.* ^39^ | 2008-2013 | Rectum | I(2), IIa(99), IIIa(11), IIIb(114), IIIc(16) | I(3), IIa(92), IIIa(11), IIIb(114), IIIc(19) | NR | NR | 242 | 239 | 57.7+11.5 | 57.2+12.1 | 156/86 | 158/81 | 137(57.1%) | 129(58.1%) | 2(0.8%) | 2(0.9%) | NR | NR | NR | NR | NR | NR | NR | NR | NR | NR |
| Stevenson A *et al.* ^59^ | 2010-2014 | Rectum | T1(18), T2(68), T3(151) | T1(11), T2(68), T3(155) | NR | NR | 238 | 235 | 65 (56-74) | 65 (56-73) | 160/78 | 151/84 | 44(18.5%) | 62(26.4%) | 1(0.4%) | 2(0.9%) | NR | NR | NR | NR | NR | NR | NR | NR | NR | NR |

**Table S4.** Time period, cancer site, disease stage, median follow-up period, patient demographics, morbidity, 30-day mortality, and overall survival (1-5 years, including median survival for each approach). MIS = minimally invasive surgery; OS = open surgery
